# Radiation oncology: Times of practice change

**DOI:** 10.3747/co.2007.143

**Published:** 2007-10

**Authors:** C.R. Freeman, E.B. Podgorsak

**Affiliations:** * Department of Radiation Oncology, McGill University Health Centre, Montreal, Quebec.; † Department of Medical Physics, McGill University Health Centre, Montreal, Quebec.

Developments in radiotherapy during the past 10–15 years, which have been driven largely by technological advances, have been nothing short of remarkable. Significant advances in tumour imaging, in radiotherapy treatment planning, and in dose delivery techniques have all contributed to improved treatment outcomes and have, in some situations, led to important changes in overall therapeutic approach.

## IMPROVED TARGET VOLUME DEFINITION

The advent of computed tomography (ct) simulation in the early 1990s was pivotal: for the first time, anatomic information could be incorporated directly into the radiotherapy treatment plan, providing much more precise identification of the boundaries of the tumour and of surrounding structures. Definitions developed by the International Commission on Radiation Units and Measurements 1,2 led to standardization of nomenclature with respect to the target volume and organs at risk, and spawned investigations to determine minimum margins that ensure adequate coverage of the tumour. In many situations, the resulting target volumes are now much different from those that would previously have constituted standard practice.

Incorporation of anatomic information acquired from magnetic resonance imaging (mri) has been immensely helpful for tumours that are better visualized using that modality—for example, central nervous system tumours, and soft tissue and bone sarcomas. Now in the era of functional imaging, positron emission tomography and magnetic resonance spectroscopy can not only add information critical for target volume definition, but can also identify areas within a tumour that may be less responsive to treatment and could be targeted to receive a higher radiation dose 3. The foregoing modalities, as well as functional mri, can also be used to identify structures that should be avoided so as to reduce the risks associated with treatment—as, for example, in patients with tumours in the central nervous system.

## BETTER TREATMENT PLANNING AND RADIATION DOSE DELIVERY

Concurrent with developments in target volume definition, tremendous progress has been made in treatment planning and radiation dose delivery techniques. For external-beam radiotherapy, conformality of dose delivery to the target volume is much more easily achieved now than in the past—using, for example, three-dimensional (3D) inverse treatment planning techniques combined with intensity-modulated dose delivery. Online treatment verification by ultrasonography or ct permits smaller safety margins to be used, which further reduces the risk of damage to normal tissues surrounding the target volume.

Improved immobilization techniques have been particularly critical in some situations: for example, stereotactic frames for patients with central nervous system tumours, or respiratory gating procedures for treatment of lung and hepatic tumours. Greater precision in dose delivery has in turn permitted safe use of dose escalation in some situations or, alternatively, much shortened fractionation schedules, such as a 20-Gy, 3-fraction schedule for patients with cancer of the lung being treated with curative intent, who in the past would have received a similar total dose, but delivered in 30 daily fractions over 6 weeks.

Important developments have also occurred in brachytherapy. Generally viewed as the ultimate conformal treatment, brachytherapy has similarly benefited from incorporation of 3D imaging into the treatment planning and dose delivery processes and from improved delivery methods. As a result, brachytherapy is now used in a larger proportion of patients than in the past, either in combination with external-beam radiotherapy or sometimes as the sole treatment (such as in patients with cancer of the rectum), replacing protracted courses of combined chemotherapy and radiotherapy.

New treatment delivery devices are coming onto the market every year, supporting and encouraging these changes in practice. Recent examples for external-beam radiotherapy include miniaturized, robotically-guided linear accelerators that adjust to the position of the tumour each day. In brachytherapy, the development of new applicators has expanded the indications for brachytherapy. Protons—whose physical properties make them extremely interesting for use in radiotherapy—are becoming more widely available.

All of these improvements have come at a cost in terms of both the equipment itself and the additional time and expertise required to use the equipment optimally. However, these additional expenses need to be evaluated in the context of savings to society achieved by improving tumour control and overall survival, or by reducing the side effects of treatment, or both. Moreover, in the context of overall expenditure for cancer care in developed countries, radiotherapy is inexpensive as compared with other treatments that have become standard over recent years—for example, hormonal treatment for men with prostate cancer and trastuzumab for women with breast cancer. The overall expenditure for radiotherapy, used in more than 50% of all patients, represents a rather small fraction of the total expenditure for cancer care. Even the newer radiation modalities such as protons or heavy ions, although requiring seemingly enormous capital outlays for infrastructure and equipment, have been estimated to be cost-effective when evaluated in these terms.

## EDUCATION, TRAINING, AND TEAMWORK

A favourable treatment outcome depends more than ever on the availability of a highly skilled team consisting of radiation oncologists, medical physicists, and radiation therapists (technologists), and on careful attention to every aspect of the treatment planning and dose delivery process. Education and training of each of these professionals is now very demanding, requiring knowledge not only of medicine but also, increasingly, sound knowledge of medical physics, technology, theory of imaging and dose delivery, and computer science. Further progress will be critically dependent on the knowledge and skills of each professional group, on how well they work together as a team, and on their collaborations with molecular biologists and industry.

## THE FUTURE

Developments in molecular biology, imaging technology, and newer delivery techniques continue apace. Already, advanced dosimetry techniques such as Monte Carlo simulation are allowing for improved integration of anatomic information into treatment planning and delivery of “personalized” treatment that is not only adapted to the clinical situation at the start of therapy, but that is also modulated if necessary during the course of treatment in response to changes in the position, size, or shape of the tumour. In the not-too-distant future, advanced imaging technologies will allow for molecular changes that take place in the tumour and surrounding normal tissues to be monitored during a course of treatment and for treatment to be modulated as appropriate to achieve maximum therapeutic effect.

These are indeed exciting times in radiation oncology, and further significant gains of major clinical relevance for patients can reasonably be anticipated over the coming years.
